# German and Roman Chamomile: Species-Specific Phytochemical Profiles, Bioactive Potential, and Relevance for Functional Foods and Nutraceutical Development

**DOI:** 10.3390/nu18132181

**Published:** 2026-07-04

**Authors:** Sebastian Such, Grzegorz Zaguła, Czesław Puchalski, Maria Czernicka

**Affiliations:** Faculty of Technology and Life Sciences, Department of Bioenergetics, Food Analysis and Microbiology, University of Rzeszów, Zelwerowicza 4 St., 35-601 Rzeszów, Poland; fenikstvit@gmail.com (S.S.); gzagula@ur.edu.pl (G.Z.); cpuchalski@ur.edu.pl (C.P.)

**Keywords:** German chamomile, Roman chamomile, apigenin, chamazulene, α-bisabolol, nobilin, phytochemicals, nutraceuticals, functional foods, food applications

## Abstract

**Background:** German chamomile (*Matricaria chamomilla* L.) and Roman chamomile (*Chamaemelum nobile* (L.) All.) are widely consumed botanical materials increasingly used in nutraceuticals and functional foods. Although both are marketed as “chamomile”, they differ in phytochemical composition, sensory profile, and potential health-promoting properties. This review compares both species as dietary and functional ingredients. **Methods**: This narrative review summarises current evidence on the botanical characteristics, phytochemical profiles, dietary forms, biological activities, safety aspects, and functional food applications of *M. chamomilla* and *C. nobile*, with emphasis on species-specific differences relevant to nutritional use and formulation. **Results:** German chamomile is more extensively characterised and is particularly rich in apigenin-related flavonoids, luteolin derivatives, α-bisabolol, and matricin-derived chamazulene, which are associated with antioxidant, anti-inflammatory, calming, gastrointestinal, and metabolic effects. Roman chamomile is distinguished by an ester-rich essential oil profile and sesquiterpene lactones, including nobilin derivatives, which contribute to its characteristic aroma and complementary biological potential. Chamomile preparations differ substantially depending on species, extraction method, processing conditions, and food matrix, indicating that infusions, extracts, powders, and essential oils are not nutritionally equivalent. **Conclusions:** German and Roman chamomile should not be treated as interchangeable botanical ingredients. Their species-specific phytochemical profiles, sensory properties, and formulation behaviour should guide their application in nutraceuticals and functional foods. Future studies should focus on bioavailability, matrix interactions, clinical validation, and improved standardisation of chamomile-derived preparations.

## 1. Introduction

Plant-derived bioactive compounds are increasingly recognised as important components of dietary strategies aimed at supporting health maintenance and reducing risk factors associated with non-communicable diseases, including metabolic syndrome, type 2 diabetes mellitus, cardiovascular disorders, oxidative stress, and chronic low-grade inflammation [1,2,3,4]. In this context, edible and traditionally consumed medicinal plants have gained renewed scientific attention, particularly when their historical use is supported by phytochemical, nutritional, pharmacological, and clinical evidence. Among such botanicals, chamomile occupies a prominent position, combining long-standing ethnobotanical relevance with widespread contemporary consumption as a herbal infusion, dietary supplement ingredient, natural flavouring agent, and functional food component [5,6,7,8,9,10,11].

The commercial and dietary term “chamomile” most commonly refers to two botanically distinct species: German chamomile, *Matricaria chamomilla* L. syn. *Matricaria recutita* L., and Roman chamomile, *Chamaemelum nobile* (L.) All. [2,12]. Although both species are commonly consumed as “chamomile tea” and are often perceived by consumers as interchangeable, they differ in botanical identity, dominant phytochemical groups, sensory profile, and the relative abundance of key bioactive constituents [13,14,15]. These differences are nutritionally and technologically relevant because they may affect extraction efficiency, stability, bioaccessibility, sensory acceptability, safety considerations, and suitability for use in functional foods, botanical supplements, nutraceutical formulations, and natural flavouring systems [5,13,14,15,16].

The relevance of chamomile-derived products is also strengthened by current consumer and market trends. A recent industry analysis reported that the global chamomile tea market reached USD 284.6 million in 2024 and is projected to reach approximately USD 501.9 million by 2033, with growth driven by increasing consumer preference for natural, caffeine-free, herbal, and wellness-oriented beverages [17]. This trend is consistent with broader consumer interest in herbal tea products shaped by health awareness, natural and organic product preferences, regional consumption habits, and growing attention to quality and safety [18]. Chamomile is particularly important in this context because it is widely recognised by consumers, traditionally associated with digestive comfort, relaxation, and mild calming effects, and increasingly investigated as an ingredient in functional beverages, enriched foods, encapsulated preparations, and nutraceutical delivery systems [5,19,20,21,22,23,24,25,26,27,28,29,30,31].

German chamomile is one of the most extensively studied medicinal and culinary plants. Its dried flower heads are included in several pharmacopoeial systems and contain a complex mixture of essential oil constituents, flavonoids, phenolic acids, coumarins, polysaccharides, amino acids, sterols, organic acids, and selected microelements [6,7,8,12,15,32,33,34,35,36,37,38]. Roman chamomile has a similarly long tradition of use, especially in European herbal practice, but remains less extensively investigated in nutritional and functional food contexts. Compared with German chamomile, it is distinguished mainly by an ester-rich essential oil profile and sesquiterpene lactones, including nobilin derivatives, which contribute to its characteristic aroma and complementary biological potential [39,40,41,42,43,44]. These species-specific profiles justify a comparative approach, while detailed phytochemical characteristics are discussed in the following sections.

Recent studies have expanded the potential use of chamomile beyond traditional infusions. Chamomile-derived powders, extracts, and essential oil preparations have been investigated as ingredients in enriched beverages, functional bakery products, fermented dairy foods, natural preservative systems, encapsulated nutraceuticals, and advanced delivery systems designed to stabilise volatile or poorly soluble compounds [19,20,21,22,23,24,25,26,27,28,29,30,31]. Such applications align with current consumer demand for natural, plant-based, minimally processed, and health-promoting food ingredients [18]. However, they also raise important questions regarding species authentication, standardisation of bioactive compounds, dose relevance, safety, sensory limitations, matrix interactions, bioavailability, and the strength of clinical evidence supporting health-related claims [16,45,46].

Despite the extensive literature on German chamomile and the long-standing traditional use of Roman chamomile, direct comparative reviews addressing both species within a unified nutritional and nutraceutical framework remain limited [5,11,47,48]. Most available studies focus either on pharmacognosy, essential oil composition, traditional medicinal uses, or individual biological activities, whereas fewer analyses integrate species-specific phytochemical differences with dietary exposure, food applications, biological activity, safety, standardisation, and formulation relevance [5]. This gap is particularly important because the commercial term “chamomile” may obscure species-specific differences relevant to consumers, researchers, food technologists, supplement manufacturers, and regulatory assessment.

Therefore, the aim of this review is to compare German chamomile (*Matricaria chamomilla* L.) and Roman chamomile (*Chamaemelum nobile* (L.) All.) as botanical ingredients for dietary, nutraceutical, and functional food applications. The review synthesises current evidence on their botanical identity, phytochemical composition, nutritional relevance, biological activities, safety aspects, regulatory status, and technological uses, with particular emphasis on how species-specific differences may influence health-promoting potential and practical applications.

## 2. Literature Search Strategy and Source Selection Criteria

This narrative review was prepared to synthesise current evidence on German chamomile (*Matricaria chamomilla* L., syn. *Matricaria recutita* L.) and Roman chamomile (*Chamaemelum nobile* (L.) All.) as botanical ingredients for nutraceutical and functional food applications. Although the review was not designed as a systematic review or meta-analysis, a structured literature search was conducted to improve transparency and reproducibility.

Relevant publications were identified using PubMed, Scopus, Web of Science, ScienceDirect, SpringerLink, Wiley Online Library, Google Scholar, and MDPI databases. The search covered studies available up to June 2026, with particular attention to publications from the last ten years. Earlier studies were included when they provided foundational botanical, phytochemical, pharmacopoeial, toxicological, or mechanistic information. Search terms included combinations of “*Matricaria chamomilla*”, “*Matricaria recutita*”, “German chamomile”, “*Chamaemelum nobile*”, “Roman chamomile”, “chamomile”, “phytochemistry”, “apigenin”, “luteolin”, “α-bisabolol”, “chamazulene”, “matricin”, “nobilin”, “essential oil”, “nutraceuticals”, “functional foods”, “dietary supplements”, “herbal infusion”, “bioavailability”, “bioaccessibility”, “safety”, “toxicity”, “allergenicity”, “drug interactions”, “standardisation”, “botanical authentication”, and “food matrix”.

The inclusion criteria comprised original research articles, reviews, pharmacopoeial monographs, regulatory documents, and relevant book chapters addressing botanical identity, phytochemical composition, dietary forms, biological activity, safety, standardisation, authentication, or food and supplement applications of German or Roman chamomile. Priority was given to studies providing species-specific data, comparative information, analytical characterisation, or evidence relevant to nutritional exposure and functional formulation. Publications were excluded when they were not available in English, lacked sufficient methodological information, focused exclusively on unrelated agronomic or ornamental aspects, or discussed chamomile only as a minor component of complex herbal mixtures without species-specific interpretation. The synthesis was organised around species-specific differences and application-relevant themes, including botanical identity, dominant phytochemical groups, preparation type, dietary relevance, bioactive potential, safety, standardisation, and functional food applications. No formal risk-of-bias assessment or quantitative quality scoring was performed, which is consistent with the narrative character of the review. However, the level of evidence was considered qualitatively, with human clinical and observational data interpreted as more directly relevant than animal, in vitro, or purely mechanistic studies.

## 3. Botanical and Phytochemical Characteristics of German and Roman Chamomile

### 3.1. German Chamomile

German chamomile (*Matricaria chamomilla* L., syn. *Matricaria recutita* L.) is one of the best-known and most widely used medicinal and dietary botanical species worldwide. Although traditionally classified as a pharmacopoeial plant, its relevance extends beyond herbal medicine owing to its broad phytochemical diversity, established safety profile, and increasing use in herbal infusions, botanical supplements, phytopharmaceutical preparations, and functional food formulations [7,8]. The genus name *Matricaria* derives from the Latin word matrix meaning “womb”, which reflects the traditional use of the plant for relieving uterine cramps and menstrual discomfort [6,12]. Historically, chamomile was used in ancient Greek, Roman, and Egyptian medicine as an anti-inflammatory, antispasmodic, sedative, and antiseptic agent, and it has also been described in Uyghur medicine since the tenth century [6,34]. *Matricaria chamomilla* belongs to the family Asteraceae and is an annual herb with an erect, branched stem reaching approximately 10–80 cm in height, finely divided leaves, and flower heads composed of white ray florets and yellow tubular disc florets [7,15]. A key diagnostic feature distinguishing *M. chamomilla* from related chamomile-like species is its hollow, conical receptacle and downward-curving white ray florets [5,8]. The pharmacopoeial raw material consists of dried flower heads, known as *Chamomillae flos*, which represent the main source of the plant’s bioactive constituents [6,15]. The species is native to southern and eastern Europe as well as western and central Asia, but it is now cultivated and naturalised on all continents. Major production areas include Hungary, Germany, Argentina, Egypt, and Poland [6,7,12].

The extensive traditional and contemporary use of German chamomile is supported by its inclusion in the pharmacopoeias of numerous countries, including the European, United States, and British pharmacopoeias [12,15]. In addition, chamomile has been classified by the United States Food and Drug Administration as Generally Recognised as Safe (GRAS) for both food and pharmaceutical applications [7,8]. This regulatory and pharmacopoeial background is important from a nutritional and nutraceutical perspective because it supports the use of chamomile-derived materials not only as traditional herbal remedies, but also as dietary botanical ingredients in infusions, dry preparations, extracts, supplements, and functional formulations. The phytochemical richness of *M. chamomilla* flowers is reflected in the presence of several major groups of bioactive constituents, including essential oil terpenoids, flavonoids, phenolic acids, coumarins, polysaccharides, sterols, organic acids, amino acids, micronutrients, and other hydrophilic and lipophilic compounds [6,8]. More than 120 constituents have been identified in chamomile essential oil, whereas the total number of characterised phytochemicals exceeds 300 compounds. This compositional diversity underlies the broad biological potential of chamomile and justifies its consideration as a nutritionally relevant botanical raw material rather than only as a classical pharmacognostic species. For clarity, the major groups of bioactive constituents identified in *M. chamomilla* flowers, together with representative compounds and their relevance to dietary, nutraceutical, and functional applications, are presented in Table 1.

The qualitative and quantitative composition of chamomile flowers is strongly influenced by chemotype, geographical origin, climate and soil conditions, developmental stage at harvest, drying procedure, storage conditions, and extraction method [12,50]. This variability has direct implications for dietary supplements and nutraceutical products, because raw material origin and processing may affect the concentration of marker compounds, sensory properties, biological activity, and formulation performance. For this reason, the phytochemical profile of chamomile should be interpreted not only as a taxonomic characteristic but also as a determinant of quality control, standardisation, and reproducibility of health-related effects.

Essential oil represents one of the key bioactive fractions of *M. chamomilla*. Its content in dried flowers generally ranges from 0.25% to 2.0%, although higher values may occasionally be reported depending on the plant material and analytical conditions [7,12]. Freshly distilled chamomile oil is characterised by a bluish-green colour associated with the presence of chamazulene [1,2]. Gas chromatography coupled with mass spectrometry (GC-MS) is commonly used to identify volatile constituents, and more than 120 terpenoid components have been reported in chamomile essential oil [6,8,51].

Sesquiterpenes are the dominant class of compounds in German chamomile essential oil. Among them, α-bisabolol and its oxides, including α-bisabolol oxide A and B, are regarded as characteristic and biologically relevant constituents. In many European chemotypes, α-bisabolol accounts for approximately 27.0–50.5% of the total essential oil fraction [7]. Chamazulene is another important compound; it is not present in the fresh plant but is formed thermally from matricin, a guaianolide sesquiterpene lactone, during steam distillation. Depending on the population and processing conditions, chamazulene may represent approximately 13.5–31.2% of the essential oil. β-Farnesene has also been reported as a dominant constituent in samples from the United States, Turkey, and Brazil, where it reached 42.59%, 25.05–30.15%, and 16.35%, respectively [7]. In Ukrainian populations, α-bisabolol oxide A may constitute as much as 65.4% of the total essential oil composition [52]. Other identified sesquiterpenes include germacrene D, spathulenol, nerolidol, β-elemene, β-bisabolene, δ-cadinene, and β-caryophyllene [12].

**Figure 1:**
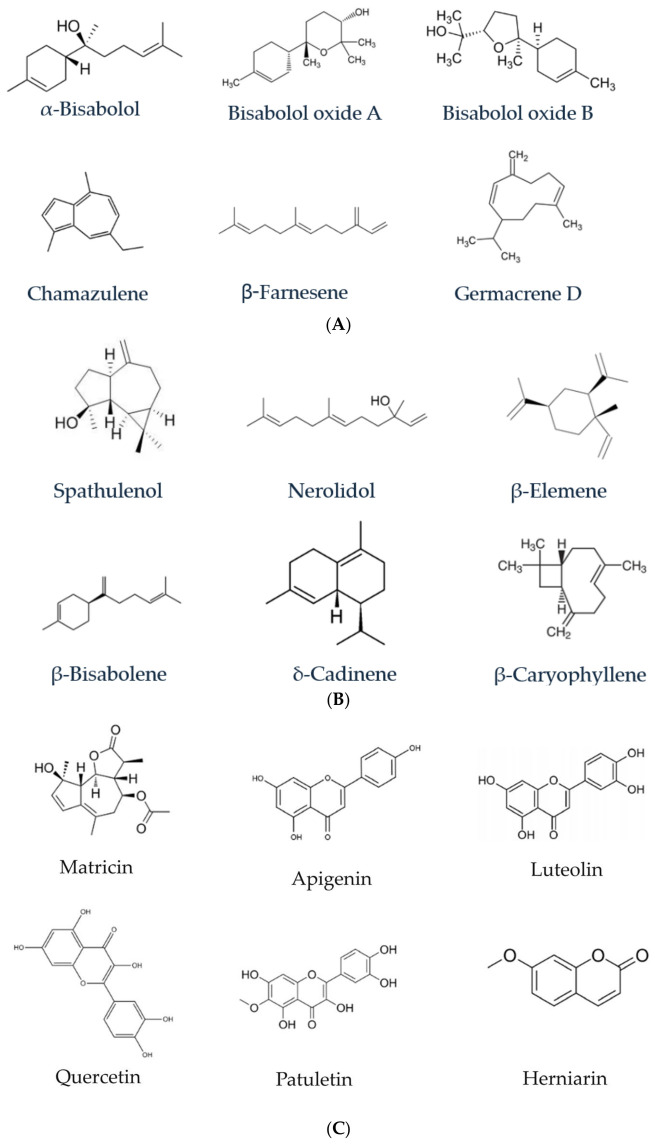
Representative chemical structures of selected bioactive constituents of *Matricaria chamomilla* L. relevant to dietary and nutraceutical applications. (**A**) Major essential oil sesquiterpenes: α-bisabolol, bisabolol oxide A, bisabolol oxide B, chamazulene, β-farnesene, and germacrene D. (**B**) Additional volatile terpenoids: spathulenol, nerolidol, β-elemene, β-bisabolene, δ-cadinene, and β-caryophyllene. (**C**) Polyphenolic and coumarin constituents: matricin, apigenin, luteolin, quercetin, patuletin, and herniarin.

Matricin deserves particular attention as a natural prochamazulene present in fresh flowers and as one of the main precursors of chamazulene formed during hydrodistillation [34]. Related guaianolide compounds, including matricarin and other guaianolides, have also been identified in chamomile flowers [9]. From a nutraceutical perspective, the essential oil fraction is relevant primarily for aroma, sensory quality, anti-inflammatory potential, and formulation properties [6,13,15]. However, because volatile constituents are sensitive to heat, oxygen, and processing conditions, their actual contribution may differ substantially among infusions, dry extracts, hydroalcoholic extracts, essential oil preparations, and encapsulated products [15,50,53]. Representative chemical structures of selected volatile, polyphenolic, and coumarin constituents of *M. chamomilla* are presented in Figure 1.

Flavonoids constitute a major group of hydrophilic polyphenolic compounds in aqueous and hydroalcoholic extracts of chamomile flowers and are among the most important bioactive constituents from the perspective of dietary exposure [6]. Approximately 50 flavonoids have been isolated and identified from *M. chamomilla*, with flavones representing the dominant subgroup. Their structural diversity results from hydroxyl and methoxyl substitutions in the aromatic rings as well as from glycosylation, which influences solubility, extractability, and potential bioavailability [6,38].

Apigenin is the principal flavone associated with chamomile flowers. It occurs predominantly in glycosylated form, especially as apigenin-7-*O*-β-D-glucoside, which is used as a quality marker in pharmacopoeial standards. The European Pharmacopoeia requires at least 0.25% apigenin-7-glucoside in the dried raw material, whereas the United States Pharmacopoeia requires at least 0.30% [15]. The apigenin aglycone has been reported at 0.25–1.22 mg/g of dry extract and is particularly concentrated in white ray florets. Apigenin-7-*O*-glucoside is quantitatively dominant, reaching 3.52–11.69 mg/g of dry extract depending on the commercial product [38]. In Estonian extracts analysed by UPLC-MS/MS, this compound reached 578.65 µg/g of dry extract [37].

Luteolin and its derivatives are also important constituents of chamomile flowers. Luteolin occurs at 0.27–2.60 mg/g of dry extract and is particularly abundant in tubular flowers and the receptacle [38]. In Estonian extracts, luteolin was detected at 310.93 µg/g [10]. Luteolin-7-*O*-glucoside may reach 4.70–9.17 mg/g of dry extract, whereas in Estonian samples it reached 1061.82 µg/g, representing the highest value among the analysed flavonoids [37,38]. Other flavonoids and related compounds identified in chamomile include quercetin, rutin, isoquercitrin, kaempferol, hispidulin, kaempferol-3-*O*-glucoside, hyperoside, patuletin, chrysoeriol, naringenin, and isorhamnetin-3-*O*-glucoside [6,37,38]. The content of flavonoids varies considerably among commercial products. Catani et al. [38] reported that total flavonoid content in six Italian commercial chamomile products ranged from 15.27 ± 0.51 to 27.31 ± 0.51 mg/g of dry extract, with apigenin and luteolin glucosides predominating over their corresponding aglycones. The glycoside-to-aglycone ratio ranged from 3:1 to 30:1 for apigenin and from 3:1 to 24:1 for luteolin. Products containing whole flower heads showed markedly higher total polyphenol content, reaching up to 100.5 ± 4.8 mg GAE/g of dry extract in an organic sample, compared with products based on sieved central flowers, which contained 41.1–62.2 mg GAE/g [38]. Sah et al. [54] reported that, within the total flavonoid fraction, apigenin, quercetin, patuletin, and luteolin accounted for 16.8%, 9.9%, 6.5%, and 1.9%, respectively, although these values were strongly dependent on variety and cultivation conditions. Importantly, apigenin concentration in *M. chamomilla* flower heads is several-fold higher than in *Chamaemelum nobile* (0.39% vs. 0.12%), which supports the preferential use of German chamomile in applications where apigenin-related activity is targeted [54].

Phenolic acids and hydroxycinnamic acid derivatives represent another nutritionally relevant group of chamomile constituents. The main compounds include chlorogenic acid, reaching up to 14.10 mg/g of dry extract, *p*-coumaric acid up to 14.80 mg/g, ferulic acid up to 3.58 mg/g, caffeic acid, and rosmarinic acid [38]. In Chinese samples analysed by HPLC, isochlorogenic acid A was dominant, with concentrations ranging from 0.1 to 5.15 mg/g, together with isochlorogenic acids B and C, neochlorogenic acid, and cryptochlorogenic acid [6]. UHPLC-HRMS analyses of Algerian chamomile flowers confirmed the presence of numerous caffeoylquinic acid derivatives, caffeoylhexosides, caffeic acid, vanillic acid, and coumaroylquinic acid derivatives [55]. These compounds contribute to the overall antioxidant potential of chamomile preparations and are particularly relevant for aqueous and hydroalcoholic extracts used in dietary and nutraceutical contexts.

Coumarins also contribute to the phytochemical profile of *M. chamomilla*. Ten coumarins have been identified in fresh flowers and leaves, including coumarin, 7-methoxycoumarin (herniarin), umbelliferone, scopoletin, isoscopoletin, esculetin, daphnetin, skimmin, daphnin, and 3,4-dihydrocoumarin [6]. Herniarin is the most abundant coumarin, reaching up to 82.79 mg/100 g of dry extract, whereas umbelliferone has been reported at up to 11.80 mg/100 g [56]. The presence of skimmin and daphnin in German chamomile was first confirmed by Petruľová-Poracká and co-workers [57]. Although coumarins are not the primary markers of chamomile quality, they contribute to the chemical complexity of the raw material and may be relevant for future studies on biological activity and safety.

In addition to polyphenols and volatile compounds, chamomile flowers contain polysaccharides, sterols, amino acids, and micronutrients. The polysaccharide content ranges from 1.29% to 3.25%; hydrolysis of these fractions yields D-galacturonic acid, D-xylose, D-galactose, L-arabinose, L-rhamnose, and D-glucose [6]. Sixteen sterols have been identified, including stigmasterol, β-sitosterol, and daucosterol [6]. Other bioactive and nutritionally relevant constituents include ascorbic acid, adenosine, γ-aminobutyric acid (GABA), choline, bitter substances, gums, and microelements such as calcium, zinc, iron, magnesium, manganese, and sodium [7]. Water-extractable amino acids complement the phytochemical profile, with proline (4.24 mg/g), alanine (3.79 mg/g), and isoleucine/leucine (2.59 mg/g) reported as notable representatives [37].

Overall, German chamomile is characterised by a phytochemical profile dominated by apigenin-related flavonoids, luteolin derivatives, phenolic acids, α-bisabolol-type sesquiterpenes, matricin-derived chamazulene, coumarins, polysaccharides, amino acids, and selected micronutrients. This composition supports its use as a dietary botanical ingredient, particularly in herbal infusions, extracts, dry preparations, and nutraceutical formulations. At the same time, the marked influence of chemotype, geographical origin, flower fraction, and processing method highlights the need for standardisation and careful quality control, especially when chamomile is used in concentrated dietary supplements rather than in traditional infusions [12,38,50].

### 3.2. Roman Chamomile

Roman chamomile (*Chamaemelum nobile* (L.) All.), also known as noble chamomile or English chamomile, is a perennial species of the Asteraceae family with a long tradition of use in European herbal medicine and dietary preparations [39,41]. The species is native to western and central Europe and Asia Minor, and its cultivation is particularly associated with England, France, Belgium, and Morocco, where it is valued for its intense aroma often described as reminiscent of fresh apples [40,42]. The dried flower heads are used as the botanical raw material. Morphologically, *C. nobile* differs from *M. chamomilla* in having a solid and relatively flat receptacle, in contrast to the hollow, conical receptacle characteristic of German chamomile [43,58]. Although both species are commonly marketed under the general name “chamomile”, *C. nobile* remains less extensively studied from phytochemical and pharmacological perspectives, and its chemical profile differs substantially from that of *M. chamomilla* [13,52]. The flowers of *C. nobile* contain several major groups of bioactive constituents, including esters of angelic, tiglic, and isobutyric acids, germacranolide-type sesquiterpene lactones, flavonoids, coumarins, phenolic acids, and other polyphenolic compounds [39,43]. The essential oil content of dried flowers ranges from approximately 0.22% during the vegetative stage to 0.80% at full flowering, as reported in hydrodistilled Croatian samples collected at six phenological stages [59,60]. In contrast to *M. chamomilla*, the essential oil of *C. nobile* is not dominated by bisabolol-type sesquiterpene alcohols; instead, it is characterised by a high proportion of aliphatic esters, while chamazulene is generally present only in marginal or trace amounts [13,52].

GC-MS analyses of essential oils obtained from Serbian and Bulgarian plant material showed that the main constituents were isobutyl angelate (28.5–35.7%), isoamyl angelate (21.1–32.5%), and other angelic acid ester derivatives [53,56]. In another study, Baranauskienė et al. [59] reported that the dominant volatile components of *C. nobile* collected at different vegetative stages included 3-methylpentyl angelate (20.11–27.56%), methallyl angelate (7.28–10.33%), isoamyl angelate (5.57–9.02%), isobutyl angelate (4.84–6.79%), 2-methylbutyl angelate (3.11–6.32%), 3-methylpentyl isobutyrate (4.29–6.64%), α-pinene (1.61–6.37%), and pinocarvone (1.46–4.67%) [59,61]. Monoterpenes such as α-pinene, camphene, sabinene, pinocarveol, and trans-pinocarveol, as well as sesquiterpenes such as β-farnesene and germacrene D, have also been identified [59,62]. This ester-rich volatile profile explains the distinctive aromatic character of Roman chamomile and clearly differentiates it from the azulene- and bisabolol-associated profile of German chamomile.

Beyond the essential oil fraction, germacranolide-type sesquiterpene lactones represent a characteristic group of secondary metabolites in *C. nobile*. Nobilin and 3-epinobilin are among the most important representatives of this group [39,41]. The total content of germacranolide sesquiterpene lactones in *C. nobile* flowers has been reported to reach up to 0.6%, which is markedly higher than in *M. chamomilla* [43,63]. Recent LC-(+)ESI/QExactive/MS/MS analyses of fresh aerial parts of *C. nobile* enabled the isolation and identification of several compounds, including scopolin, tuberonic acid glucoside, phenylethyl β-D-glucopyranoside, 1-methylpropyl β-D-glucopyranoside, apigenin-7-*O*-rutinoside, apigenin, hydroxyisonobilin, 3-epihydroxyisonobilin, nobilinone A, nobilin, genkwanin, and newly described nobilin-related derivatives [13,42]. Among them, 11,13-dihydro-8-tigloylhydroxyisonobilin was reported as a previously undescribed nobilin derivative and was structurally characterised using NMR and high-resolution mass spectrometry.

Flavonoids constitute another relevant group of constituents in Roman chamomile. Apigenin occurs both as a free aglycone and in glycosylated forms, including apigenin-7-*O*-rutinoside and apigenin-7-*O*-glucoside, and has been proposed as a chemical marker of the species [48,49]. Apigenin and its glycosidic derivatives have been identified as major polyphenolic constituents in Roman chamomile flower tea [8,64]. Other flavonoids reported in *C. nobile* include luteolin, quercetin, patuletin, and chrysoeriol [13,43]. A specific flavonoid glycoside, chamaemeloside, was also isolated from *C. nobile* and described as a characteristic compound of this species [43,65]. However, the overall apigenin content of Roman chamomile is lower than that of German chamomile, which is an important distinction when selecting raw material for products targeting apigenin-related nutraceutical effects [54].

**Figure 2:**
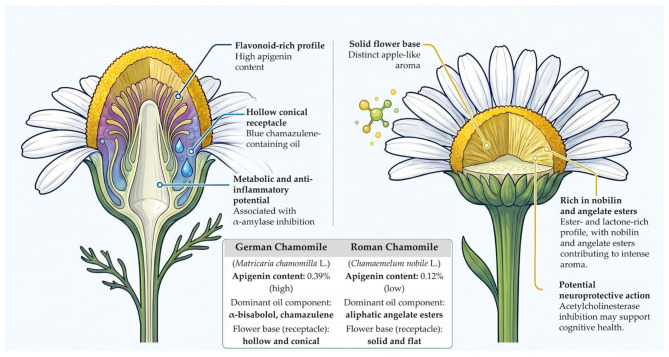
Comparative overview of German and Roman chamomile, highlighting key botanical, phytochemical, biological, and dietary/nutraceutical differences between *Matricaria chamomilla* L. and *Chamaemelum nobile* (L.) All.

Coumarins further contribute to the chemical profile of Roman chamomile. The main representatives include scopolin, a glycoside of scopoletin, and free scopoletin, while herniarin and umbelliferone have also been detected in aerial parts of the plant [42,57,66]. These compounds are shared, at least in part, with *M. chamomilla*, but their relative abundance and coexistence with the ester-rich essential oil and nobilin-containing lactone fraction contribute to a distinct phytochemical profile. Collectively, *C. nobile* can be regarded as an ester- and lactone-rich chamomile species with a pronounced aromatic character, whereas *M. chamomilla* is more strongly associated with apigenin-related flavonoids, bisabolol-type sesquiterpenes, and matricin-derived chamazulene. The key botanical and phytochemical differences between German and Roman chamomile are shown schematically in Figure 2.

Although both species are commonly referred to as “chamomile”, they differ in botanical identity, dominant phytochemical groups, sensory profile, and practical relevance. These key differences are compared in Table 2.

In summary, German and Roman chamomile should not be treated as interchangeable botanical ingredients. Their species-specific phytochemical profiles, sensory properties, and dominant bioactive constituents should guide the selection of raw materials for infusions, extracts, dietary supplements, and functional food formulations.

## 4. Dietary Forms and Nutraceutical Relevance

Chamomile-derived preparations are consumed and formulated in several forms, ranging from traditional herbal infusions to concentrated extracts, powders, essential oils, and encapsulated systems. These forms differ substantially in phytochemical composition, extractability of bioactive compounds, sensory properties, stability, and potential relevance for dietary or nutraceutical applications. Therefore, the nutritional significance of chamomile depends not only on the botanical species used, but also on the preparation type, extraction medium, processing conditions, and final product matrix.

Herbal infusions remain the most traditional and widespread dietary form of chamomile consumption. Infusions prepared from dried flower heads of *Matricaria chamomilla* are among the most commonly consumed herbal beverages worldwide, either as single-ingredient teas or as components of herbal blends [34]. A typical preparation involves pouring boiling water over dried flowers, usually followed by several minutes of infusion in a covered vessel to reduce the loss of volatile constituents [7]. Chamomile infusion contains only part of the essential oil fraction but extracts a relevant proportion of hydrophilic constituents, including flavonoids and phenolic acids. It has been reported that approximately 10% of essential oil constituents and about 29–30% of flavonoids present in the flowers may pass into the infusion [67]. This makes chamomile tea a particularly important form of actual dietary exposure to water-extractable bioactive compounds.

The relevance of chamomile infusions as dietary sources of bioactive compounds is further supported by studies on the bioaccessibility of flavonoids during digestion. Although the total amount of apigenin and related flavones ingested with herbal tea is lower than in concentrated extracts, aqueous preparations may provide physiologically meaningful exposure to water-extractable polyphenols [68]. Recent in vitro digestion data indicate that apigenin released from chamomile tea may show favourable bioaccessibility, suggesting that traditional infusions should not be considered merely as mild sensory beverages but also as relevant dietary carriers of flavonoid compounds. Nevertheless, the actual bioavailability of apigenin and other chamomile-derived polyphenols depends on glycosylation pattern, food matrix, gastrointestinal stability, metabolism, and individual digestive conditions [69].

Roman chamomile (*Chamaemelum nobile*) is also traditionally consumed as an infusion, especially in Mediterranean and western European countries, including England, France, and Belgium [39,43]. Compared with German chamomile, Roman chamomile tea is characterised by a more intense apple-like aroma, which reflects the predominance of angelic and isobutyric acid esters in its essential oil fraction [40,63]. Carnat et al. [64] described the aromatic and polyphenolic composition of *C. nobile* flower infusion, identifying apigenin, its glycosides, and other flavonoids as the main health-relevant polyphenolic constituents transferred into the aqueous preparation [13,64]. Thus, although both species are consumed as “chamomile tea”, their sensory profile and phytochemical contribution to the infusion differ markedly.

Beyond traditional infusions, chamomile is used in the form of aqueous, hydroalcoholic, and ethanolic extracts. Aqueous extracts are compatible with beverages and other hydrophilic food or supplement matrices, but they are less efficient in extracting lipophilic essential oil constituents. Hydroalcoholic and ethanolic extracts, in contrast, may provide higher concentrations of selected polyphenols and less polar bioactive constituents, which makes them relevant for botanical concentrates, liquid preparations, and dietary supplements [7,8,15]. These extracts may be further processed into dry extracts or powders intended for capsules, tablets, instant herbal mixtures, or other nutraceutical formulations.

Plant powders and whole-flower preparations represent another important form of chamomile use. They preserve a broader spectrum of constituents present in the raw material, including polyphenols, fibre-like fractions, and selected minerals, but their composition may vary depending on flower fraction, particle size, storage conditions, and processing method [12,50]. From a formulation perspective, powders are convenient for dosing and incorporation into dry supplement systems; however, they may pose challenges related to dispersibility, stability, aroma retention, and sensory acceptability.

From a nutraceutical perspective, the transition from traditional infusion to concentrated extract changes both the expected bioactive exposure and the safety profile of the preparation. Dry and hydroalcoholic extracts may concentrate flavonoids, phenolic acids, and selected less polar constituents, but they also require more precise standardisation than herbal teas [70]. This is particularly important for preparations marketed as capsules, tablets, liquid botanical supplements, or standardised extracts, where reproducible content of marker compounds, such as apigenin derivatives or essential oil constituents, is necessary to ensure batch-to-batch consistency and meaningful comparison of biological effects [39,71].

Essential oils obtained from German or Roman chamomile constitute a more concentrated and chemically distinct form of chamomile-derived material. In *M. chamomilla*, the essential oil is associated mainly with α-bisabolol, bisabolol oxides, chamazulene, and related sesquiterpenes, whereas in *C. nobile* it is characterised by a high proportion of angelate and tiglate esters [7,8,13,52]. Essential oils are relevant for flavouring, aromatherapeutic, cosmetic, and selected nutraceutical applications, but their use requires greater attention to dose, stability, sensory intensity, and safety than traditional infusions. This distinction is particularly important because essential oils cannot be considered nutritionally equivalent to aqueous teas or mild botanical preparations. Encapsulation technologies have been investigated as strategies to improve the stability, bioavailability, sensory acceptability, and controlled release of chamomile essential oils and extracts [19]. Chamomile essential oil and extracts have been encapsulated using various carrier systems, including silica nanoparticles, silver nanoparticles, chitosan-based nanocarriers, and alginate microcapsules [20,61,72]. Such approaches may help protect volatile constituents from oxidation, heat, and evaporation, while also reducing bitterness or excessive aroma intensity and improving compatibility with food or supplement matrices [19,73]. In the context of nutraceutical development, encapsulation is particularly relevant for concentrated chamomile preparations, where stability and reproducible delivery of bioactive compounds are critical.

The choice of chamomile-derived form should therefore be aligned with the intended dietary or nutraceutical function. Traditional infusions are most relevant for daily dietary exposure and consumer-friendly use, aqueous extracts for beverage and hydrophilic systems, hydroalcoholic extracts for concentrated botanical preparations, powders for dry supplement formulations, essential oils for aroma-rich and highly concentrated applications, and encapsulated systems for improved stability and controlled delivery. The main practical forms of chamomile-derived preparations are highlighted in Table 3.

## 5. Health-Promoting Properties Relevant to Nutritional Use

The health-promoting potential of chamomile preparations results from the combined activity of several phytochemical groups, including flavonoids, phenolic acids, sesquiterpenes, coumarins, and, in the case of Roman chamomile, germacranolide-type sesquiterpene lactones. Evidence from in vitro, in vivo, and selected clinical studies indicates that chamomile flowers and extracts may exert antioxidant, anti-inflammatory, antimicrobial, anxiolytic, metabolic, antispasmodic, and gastroprotective effects [6,50,74,75,76]. In the context of nutritional use, these effects should be interpreted primarily as supportive and health-maintenance properties rather than as direct therapeutic claims, especially when chamomile is consumed as an infusion or mild botanical preparation.

**Figure 3:**
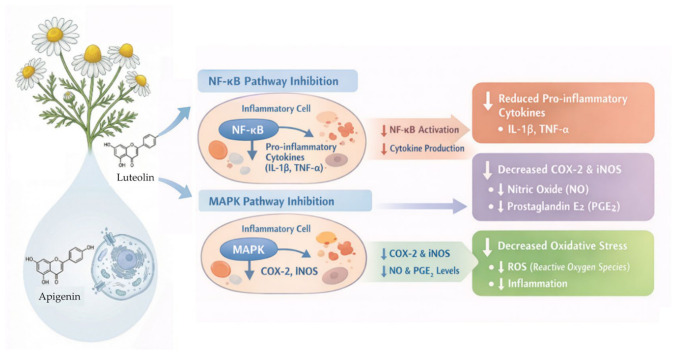
Schematic representation of the proposed molecular mechanism underlying the anti-inflammatory activity of flavonoids from *Matricaria chamomilla* L.

Anti-inflammatory activity is among the best documented biological properties of *Matricaria chamomilla*. This activity is attributed to the synergistic contribution of several constituent groups, particularly flavonoids and essential oil components [6]. Chamomile flavonoids, especially apigenin and luteolin, have been shown to inhibit the NF-κB signalling pathway, thereby reducing the expression of pro-inflammatory mediators such as TNF-α, IL-1β, IL-6, and inducible nitric oxide synthase (iNOS) [54]. Catani et al. [38] demonstrated that chamomile extracts reduced reactive oxygen species (ROS) formation in Caco-2 enterocytes exposed to a pro-oxidant stimulus, with stronger effects observed in samples richer in flavonoids. The proposed molecular mechanism underlying the anti-inflammatory activity of chamomile flavonoids is shown in Figure 3.

The essential oil fraction also contributes to the anti-inflammatory potential of German chamomile. Chamazulene has been reported to inhibit cyclooxygenase-2 (COX-2) and 5-lipoxygenase (5-LOX), thereby limiting the synthesis of prostaglandins and leukotrienes, which are key mediators of the inflammatory response [77]. α-Bisabolol has additionally been reported to modulate the release of pro-inflammatory cytokines and to exert protective effects against acute liver injury in experimental models [78]. Apigenin may further attenuate inflammation through the GSK-3β/Nrf2 signalling pathway in LPS-stimulated BV2 microglial cells [77]. Chamomile extracts and essential oils also exhibit antioxidant activity, as demonstrated using standard assays such as DPPH, ABTS, FRAP, CUPRAC, and lipid peroxidation inhibition tests [6]. In recent studies, ethanolic extracts of *M. chamomilla* flowers showed DPPH radical-scavenging activity with an IC_50_ value of 13.15 ± 0.95 µg/mL, whereas flower infusion showed an IC_50_ value of 24.46 ± 0.35 µg/mL [30]. Antioxidant activity is closely associated with total flavonoid and polyphenol content, particularly with the presence of apigenin and luteolin derivatives, which are linked to higher ABTS and FRAP values [11]. At the cellular level, these flavonoids may activate the Nrf2 pathway and induce endogenous antioxidant enzymes, including superoxide dismutase, catalase, glutathione peroxidase, and glutathione reductase [54]. Luteolin and luteolin-7-*O*-glucoside have also been shown to increase reduced glutathione (GSH) levels in enterocytes, thereby supporting cellular antioxidant defence [38].

The antimicrobial potential of *M. chamomilla* has been reported against Gram-positive bacteria, including *Staphylococcus aureus* MRSA and *Bacillus subtilis*, as well as Gram-negative bacteria, including *Pseudomonas aeruginosa* and *Escherichia coli* [6]. Essential oil generally shows stronger antimicrobial activity than aqueous or alcoholic extracts, which is consistent with the membrane-disruptive action of lipophilic constituents such as bisabolol and chamazulene. The antifungal peptide MCh-AMP1 isolated from *M. chamomilla* has shown broad-spectrum activity against *Candida albicans*, increasing membrane permeability and inducing lethal ROS production [6,79]. In food microbiology, the hexane extract of *M. chamomilla* was reported to act synergistically with nisin against *Alicyclobacillus acidoterrestris*, a spoilage microorganism relevant to fruit juices [80]. These findings are particularly relevant to functional food and preservation-oriented applications, although their direct nutritional implications require careful interpretation.

**Figure 4:**
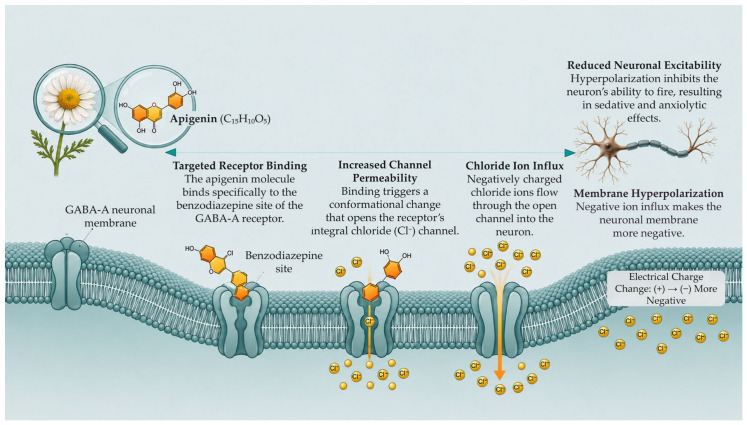
Proposed sedative and anxiolytic mechanism of action of apigenin from chamomile.

The anxiolytic and sedative effects of chamomile are mainly associated with apigenin, a flavone capable of binding to GABA-A receptors at the benzodiazepine site. This interaction may increase chloride channel permeability, leading to membrane hyperpolarisation and reduced neuronal excitability, as illustrated in Figure 4 [77]. Selected clinical evidence also supports the calming relevance of chamomile preparations. In a double-blind clinical trial involving cancer patients undergoing chemotherapy, regular consumption of chamomile infusion significantly reduced depression scores from 25.81 to 19.49 points and decreased anxiety scores (*p* < 0.05) [81]. Mild sedative effects may also be supported by selected volatile constituents of the essential oil, including esters such as isoamyl isobutyrate and isobutyl isobutyrate [6].

Chamomile preparations are also relevant to gastrointestinal and metabolic health. Extracts of *M. chamomilla* may inhibit α-amylase and α-glucosidase, enzymes involved in starch and carbohydrate digestion, suggesting a potential role in moderating postprandial glycaemia [6]. Kerbab et al. [30] reported that chamomile infusion exhibited strong α-amylase inhibitory activity, with an IC_50_ value of 11.27 µg/mL. Chamomile flavonoids may additionally inhibit aldose reductase and protein glycation, processes implicated in diabetes-related complications [54]. Animal studies further indicate reductions in fasting blood glucose and improvements in metabolic parameters following regular administration of chamomile extracts [82]. Although these findings are promising, they should be interpreted as supportive evidence for metabolic modulation rather than as proof of therapeutic efficacy in humans.

The traditional gastrointestinal use of chamomile is supported by its antispasmodic and gastroprotective properties. Chamomile extracts exert relaxing effects on gastrointestinal smooth muscle, partly through potassium channel activation, which may explain their traditional use in alleviating intestinal cramps, colic, and symptoms associated with irritable bowel syndrome [6]. Additional mechanisms include antagonism of acetylcholine and histamine, leading to smooth muscle relaxation [83,84]. α-Bisabolol, one of the main essential oil constituents, has been associated with gastroprotective effects through reduction in gastric acid secretion and direct protection of the gastric mucosa against irritating factors [54,78,85,86].

Preclinical studies have also reported cytotoxic and antiproliferative effects of selected chamomile constituents, particularly apigenin, luteolin, and α-bisabolol, against different cancer cell lines [6]. Apigenin has been reported to inhibit proliferation of HepG2 and HL-60 cells through modulation of the Wnt/β-catenin pathway, regulation of cyclins, and increased expression of p53, p21, and pro-apoptotic factors [77]. α-Bisabolol inhibited migration and invasion of glioma cells by downregulating c-Met receptor expression [78]. α-Bisabolol oxide A and apigenin-7-*O*-glucoside inhibited migration of Caco-2 colon cancer cells and affected VEGFR2-related angiogenic signalling [87], while hydroalcoholic extracts of aerial parts of chamomile showed dose- and time-dependent pro-apoptotic and antiproliferative effects against MCF-7 and MDA-MB-468 breast cancer cell lines [88]. These findings are mechanistically interesting but remain primarily preclinical and should not be translated into health claims without clinical validation.

Roman chamomile (*Chamaemelum nobile*) shows a partly distinct biological profile, reflecting its different phytochemical composition. Germacranolide-type sesquiterpene lactones, which are more characteristic of *C. nobile* than *M. chamomilla*, contain reactive α,β-unsaturated lactone groups capable of covalently interacting with sulfhydryl groups of target proteins [39,89]. This mechanism may contribute to their anti-inflammatory properties. Alkylation of cysteine residues in the IKKβ subunit of IκB kinase may prevent IκB phosphorylation and degradation, thereby inhibiting NF-κB activation and reducing the expression of pro-inflammatory genes such as TNF-α, IL-1β, IL-6, COX-2, and iNOS [90,91].

In vivo studies further support the anti-inflammatory and analgesic potential of *C. nobile*. Aremu et al. [92] reported that essential oil from *C. nobile* flowers administered at 180 mg/kg comparable to those of ibuprofen at 100 mg/kg in rat models of egg albumin-induced inflammation, formalin testing, and writhing response. Analgesic activity of the oil was also confirmed in chemical and thermal pain models in mice and rats. Batovska et al. [48] reported that essential oils from Bulgarian *C. nobile* showed stronger inhibition of albumin denaturation than *M. chamomilla* oils and effects comparable with prednisolone, suggesting a marked anti-inflammatory potential of Roman chamomile [48,93].

The antioxidant activity of *C. nobile* extracts has been demonstrated in vitro using standard assays [13,59]. Aqueous extracts from flowers collected at full flowering showed FRAP values up to 650 µmol TE/g and ORAC values up to 5601 µmol TE/g dry extract [48,54]. However, ABTS activity of *C. nobile* extracts may be lower than that of *M. chamomilla*, possibly owing to lower levels of chamazulene and polyphenolic flavonoids [48,52]. At the cellular level, flavonoids such as apigenin and luteolin may activate the Nrf2 pathway and increase the expression of endogenous antioxidant enzymes, including SOD, catalase, and GSH-Px [27,43].

The essential oil of Roman chamomile has also shown antimicrobial activity in disk diffusion and MIC assays [61,94]. Aćimović et al. [95] reported strong antibacterial activity of steam-distilled essential oil from Serbian *C. nobile* plants, with inhibition zones reaching 40 mm against *Staphylococcus aureus*, *Enterococcus faecalis*, *Salmonella enterica* Typhimurium, and *Pseudomonas aeruginosa*, whereas hydrolate from the same plant showed no significant activity [48,95]. The antimicrobial mechanism of angelate ester-rich oils is thought to involve disruption of cytoplasmic membrane integrity and disturbance of the proton gradient, leading to bacterial cell lysis [96,97]. Anthelmintic activity has also been reported for *C. nobile* oil rich in isobutyl angelate, with IC_50_ values of 0.842 mg/mL in the egg hatch inhibition assay and 0.117 mg/mL in the larval development assay against *Haemonchus contortus* [98,99,100].

Preclinical cytotoxic and pro-apoptotic activity has also been described for *C. nobile*. An ethyl acetate extract showed antiproliferative activity against MCF-7 breast cancer cells, K562 leukaemia cells, and SKMEL-3 melanoma cells, with reported IC_50_ values of 0.002, 0.04, and 0.04 mg/mL, respectively, and relative selectivity compared with normal gingival fibroblasts [63,101]. The proposed mechanism involved mitochondrial apoptosis, increased Bax expression, reduced Bcl-2 expression, an increased Bax/Bcl-2 ratio, and cell-cycle blockade in the G2/M phase [101,102]. Recent studies on isolated flavonoids and sesquiterpene lactones from *C. nobile* further showed tyrosinase inhibitory activity, with apigenin-7-*O*-rutinoside displaying an IC_50_ value of 32.09 µM, stronger than kojic acid used as a reference compound [42,103]. The same compounds also inhibited acetylcholinesterase, with IC_50_ values ranging from 181.58 to 387.99 µM, the highest activity being reported for apigenin-7-*O*-rutinoside and santosetin [42,104,105]. These results suggest potential neurophysiological relevance, although clinical confirmation is still required.

Taken together, both German and Roman chamomile exhibit health-relevant biological activities that may support their use as dietary botanicals and nutraceutical ingredients. *M. chamomilla* is more strongly supported by data on flavonoid-rich preparations, apigenin-related mechanisms, antioxidant activity, metabolic modulation, and gastrointestinal comfort. *C. nobile*, in contrast, appears particularly relevant for its ester-rich essential oil and germacranolide lactone profile, which may contribute to anti-inflammatory, antimicrobial, aromatic, and neurophysiological effects. However, many of the reported effects, especially anticancer, antiparasitic, and enzyme-inhibitory activities, are based mainly on preclinical models; therefore, their nutritional relevance depends on preparation type, dose, bioavailability, and the availability of human evidence.

## 6. Safety, Interactions and Standardisation Issues

Although German and Roman chamomile are widely consumed and generally regarded as safe botanical materials, their use in dietary, nutraceutical, and functional food applications requires careful consideration of preparation type, dose, consumer group, evidence level, and product standardisation [6,7,8,12,15,106]. The overall safety profile of chamomile is favourable, particularly when the plant is consumed as a traditional herbal infusion at customary dietary doses [6,7,8,50]. *Matricaria chamomilla* has been included in several pharmacopoeial and herbal monograph systems and is recognised in food-related regulatory frameworks [7,8,15,67,106,107]. Similarly, *Chamaemelum nobile* and selected preparations derived from Roman chamomile are used as natural flavouring materials and are included in regulatory contexts relevant to food and flavouring applications [106,108,109]. However, safety conclusions derived from traditional tea consumption should not be automatically extrapolated to concentrated extracts, essential oils, encapsulated systems, or high-dose dietary supplements [6,15,50,110].

The form of preparation is a major determinant of exposure and risk. Aqueous infusions provide relatively mild exposure to water-soluble flavonoids, phenolic acids, and limited amounts of volatile compounds [7,8,106]. In contrast, hydroalcoholic extracts, dry extracts, essential oils, and encapsulated formulations may concentrate specific groups of bioactive constituents and thereby alter both biological activity and the safety profile [15,19,20,53,61,73]. Essential oils are particularly important in this regard because they contain highly concentrated volatile compounds and may produce pronounced sensory and biological effects even at low doses [6,13,52]. Therefore, essential oil preparations should not be considered nutritionally equivalent to chamomile teas, and their use in food, supplement, or aromatherapeutic products requires stricter control of dose, purity, stability, and labelling [6,50,108,109].

A critical issue in safety assessment is the distinction between well-documented risks, plausible but less substantiated interactions, and mainly theoretical concerns. The strongest clinical relevance is associated with allergic reactions, particularly in individuals sensitised to plants of the Asteraceae/Compositae family [6,7,8,50,52,67,107]. Because both German and Roman chamomile belong to this family, cross-reactivity may occur in consumers allergic to ragweed, chrysanthemum, marigold, asters, daisies, or related species. Reported reactions include contact dermatitis, urticaria, oral allergy symptoms, bronchospasm, throat swelling, and, rarely, anaphylaxis [6,9,50,52]. Although severe reactions appear uncommon, they are clinically relevant in sensitised individuals and may be more likely after mucosal exposure, topical use, or consumption of concentrated preparations [9,50,57]. Importantly, some historical reports of “chamomile” allergy may also have been confounded by adulteration or substitution with more allergenic chamomile-like species, such as *Anthemis cotula*, which highlights the importance of botanical authentication [45,52,111].

Drug interactions should be interpreted according to the strength of available evidence. The possible interaction with anticoagulant or antiplatelet therapy is among the most practically relevant concerns because it is supported by case-based evidence, pharmacological plausibility, and the presence of flavonoids and coumarin-related compounds in chamomile preparations [6,10,12,13,36,37,38,111]. Caution is therefore advisable in individuals using warfarin, aspirin, clopidogrel, or other agents affecting haemostasis, particularly when high-dose extracts, polyherbal products, or long-term supplementation are used. Discontinuation of concentrated chamomile preparations before surgery is often recommended in herbal safety resources [36,37,112,113]. By contrast, interactions with benzodiazepines, opioids, sedative antidepressants, antiepileptic drugs, alcohol, or other central nervous system-active agents are based mainly on pharmacological plausibility and preclinical or mechanistic evidence rather than robust clinical documentation [6,37,38,77]. Similar caution applies to potential additive effects with antidiabetic or antihypertensive medications, where the concern is biologically plausible in view of reported glycaemia- and blood-pressure-modulating effects, but the clinical magnitude of such interactions remains insufficiently defined [36,37,82]. Weak estrogen-like activity has also been discussed for some chamomile constituents; however, this concern is currently supported mainly by limited experimental evidence and should be presented as a precaution rather than a confirmed clinical risk [6,36,38]. The main safety considerations for German and Roman chamomile in dietary, nutraceutical, and functional food applications are summarized in Table 4.

Special population groups require additional attention. Pregnant and lactating women, infants, young children, elderly consumers, and patients with chronic diseases should use concentrated chamomile preparations cautiously unless safety has been established for the specific formulation and dose [6,50,52,114,115]. Although mild infusions are widely consumed, but this does not necessarily provide evidence for the safety of standardised extracts, essential oils, or encapsulated formulations in vulnerable populations. Regulatory and herbal monograph sources generally distinguish between traditional oral use and insufficient evidence for pregnancy and lactation. Therefore, a clear distinction between dietary use, supplement use, and medicinal-dose preparations is essential [50,110]. In practical terms, occasional consumption of mild chamomile infusion is not equivalent to repeated intake of concentrated extracts or essential oil-containing products, and vulnerable consumers should be specifically considered in labelling and risk communication.

Standardisation remains a major challenge for chamomile-derived products. The concentration of bioactive constituents may vary according to species, chemotype, geographical origin, cultivation conditions, plant part, developmental stage at harvest, drying method, storage, extraction solvent, and processing technology [12,15,38,50,71,120,121,122]. In *M. chamomilla*, apigenin-7-*O*-glucoside is an important pharmacopoeial quality marker, whereas essential oil quality is often associated with α-bisabolol, bisabolol oxides, matricin-derived chamazulene, and related sesquiterpenes [6,9,15]. In *C. nobile*, quality assessment should take into account the ester-rich essential oil profile, particularly angelate and tiglate esters, as well as nobilin-containing sesquiterpene lactone fractions. Because the two species differ markedly in their phytochemical fingerprints, they should not be treated as interchangeable raw materials in standardised supplements, functional foods, or nutraceutical formulations [13,42,43]. Current quality-control approaches should go beyond single-marker standardisation. Chromatographic fingerprinting, untargeted and targeted metabolomics, essential oil profiling, and chemometric classification can provide a more comprehensive assessment of batch-to-batch consistency, species-specific composition, and processing-related variability [15,45,52]. DNA barcoding and other molecular authentication tools may further support the identification of raw materials, particularly when morphological features are lost during drying, powdering, extraction, or encapsulation. Such approaches are especially relevant for commercial products labelled generically as “chamomile”, which may not clearly distinguish between *M. chamomilla*, *C. nobile*, and other chamomile-like species [45,52,123]. A lack of botanical precision can complicate safety assessment, clinical interpretation, pharmacovigilance, and consumer communication [110].

Contamination and adulteration also require explicit attention in the safety assessment of chamomile ingredients. As with other botanical raw materials, chamomile flowers and derived preparations may be affected by heavy metals, pesticide residues, mycotoxins, microbiological contamination, foreign plant material, inappropriate drying, or poor storage conditions [116,117,118,119]. These hazards are not specific to chamomile alone, but they are highly relevant for nutraceuticals, functional foods, teas, and dietary supplements because such products are often perceived by consumers as inherently safe. Quality control should therefore include not only marker-compound quantification but also testing for contaminants, compliance with food and supplement regulations, and verification of botanical identity. This is particularly important for concentrated extracts and encapsulated formulations, where contaminants or adulterants may also become concentrated together with desired bioactive compounds.

From a regulatory perspective, chamomile-derived products fall at the interface between foods, herbal medicinal products, dietary supplements, flavouring agents, cosmetics, and nutraceutical preparations [9,106,108,109,110]. This creates challenges for dose justification, health claim substantiation, labelling, safety assessment, and benefit–risk evaluation. Existing pharmacopoeial and herbal monograph specifications provide useful quality parameters, but they do not always predict biological efficacy, adverse-event profiles, or clinical outcomes. Moreover, many over-the-counter products differ in galenic form, extraction solvent, marker compound content, and declared dose, which limits comparability across studies and products [9,15,50,110].

Taken together, safety assessment of German and Roman chamomile should be formulation-specific and evidence-based. Traditional infusions represent the mildest and best-established dietary form, whereas concentrated extracts, essential oils, encapsulated systems, and high-dose supplements require stricter control of species identity, dose, purity, contaminants, labelling, and standardisation [6,9,19,20,50,53,61,73,110]. Therefore, product evaluation should not rely on the generic term “chamomile”, but should consider botanical species, preparation type, intended use, consumer group, and quality-control framework.

## 7. Limitations

Several limitations should be considered when interpreting the findings of this review. The manuscript was designed as a narrative review aimed at integrating botanical, phytochemical, nutritional, nutraceutical, functional food, and safety-related perspectives on German and Roman chamomile. Accordingly, it was not based on a registered protocol, systematic search strategy, or formal evidence-grading framework. This approach allowed a broad and interpretative synthesis of the topic, but the literature was not selected and assessed according to the procedures used in systematic reviews.

The methodological quality and risk of bias of individual studies were not formally evaluated. Therefore, the strength of evidence supporting specific biological effects, functional applications, and safety considerations should be interpreted cautiously, especially when conclusions are based on preclinical, mechanistic, or formulation-specific studies rather than controlled human trials.

The available literature is also unevenly distributed between the two species. Research on *Matricaria chamomilla* is considerably more abundant than research on *Chamaemelum nobile*, particularly with regard to phytochemical characterisation, biological activity, and human evidence. This imbalance may influence comparative interpretation and limits the possibility of drawing equally strong conclusions for both species.

Finally, comparisons across studies are complicated by substantial phytochemical variability related to geographical origin, genotype, chemotype, cultivation conditions, harvest stage, processing, extraction method, and formulation technology. Differences in analytical methods, dose reporting, marker compounds, and preparation types further limit reproducibility and cross-study comparability. Consequently, phytochemical differences between the two species should not be assumed to translate automatically into equivalent clinical differences.

## 8. Future Perspectives

Future research should prioritise well-designed human clinical trials using clearly defined, chemically characterised, and standardised chamomile preparations. Direct comparative studies between German and Roman chamomile would be particularly valuable to determine whether their phytochemical differences translate into clinically meaningful differences in physiological effects, tolerability, sensory properties, and nutritional relevance. Such studies should include transparent dose reporting, appropriate control groups, clinically relevant endpoints, and assessment of dose–response relationships. Improved reproducibility also requires harmonised extraction procedures and phytochemical markers. In German chamomile, apigenin derivatives and bisabolol-type sesquiterpenes remain important quality indicators, whereas Roman chamomile requires greater attention to ester-rich essential oil profiles and sesquiterpene lactones. Future quality control should move beyond single-marker approaches and integrate chromatographic fingerprinting, metabolomics, chemometrics, and DNA-based botanical authentication to distinguish species, chemotypes, adulterants, and processing-related variability.

Further studies should clarify the bioavailability, metabolism, gastrointestinal transformation, gut microbiota interactions, and food matrix effects of chamomile-derived compounds. These aspects are essential because biological activity depends not only on the concentration of bioactives in the raw material, but also on their release, stability, absorption, and transformation within beverages, dairy products, bakery products, encapsulated supplements, and other functional food systems. Advanced omics approaches, including metabolomics, nutrigenomics, transcriptomics, and microbiome-oriented analyses, may help identify species-specific mechanisms and biomarkers of response. Innovative delivery systems, including nanoencapsulation, microencapsulation, emulsions, and controlled-release formulations, also warrant further investigation to improve the stability, bioavailability, sensory acceptability, and efficacy of chamomile bioactives. However, the development of such formulations should be accompanied by stronger clinical evidence, formulation-specific safety assessment, contaminant monitoring, and species-specific labelling. This is particularly important because traditional use as an infusion cannot be directly extrapolated to concentrated extracts, essential oils, encapsulated systems, or high-dose supplements.

## 9. Conclusions

German chamomile (*Matricaria chamomilla* L.) and Roman chamomile (*Chamaemelum nobile* (L.) All.) represent closely related but phytochemically and functionally distinct botanical resources with relevance for modern nutrition, nutraceuticals, and functional food design. Although both species are traditionally consumed as herbal infusions and are often treated interchangeably in commercial practice, this review shows that they differ in dominant bioactive constituents, sensory attributes, preparation profiles, and potential applications. German chamomile appears particularly promising for dietary and nutraceutical applications related to oxidative stress, inflammation, metabolic balance, and mild stress-related conditions, mainly due to its apigenin-related flavonoids and bisabolol-type sesquiterpenes. Roman chamomile offers distinctive potential associated with its ester-rich essential oil profile and sesquiterpene lactones, including nobilin derivatives, which may support sensory, flavour-oriented, and selected neuroactive applications. However, these interpretations should be regarded as promising rather than definitive, because phytochemical differences do not necessarily translate into equivalent clinical differences.

The future use of chamomile in functional foods and nutraceuticals should therefore be based on a balanced integration of traditional knowledge, phytochemical evidence, safety assessment, formulation science, and clinical validation. Wider application in beverages, dairy products, bakery goods, encapsulated nutraceuticals, and advanced delivery systems is scientifically plausible, but requires authenticated raw material, clear species identification, standardised phytochemical markers, contaminant control, and formulation-specific safety evaluation.

In summary, German and Roman chamomile should not be treated as interchangeable botanical ingredients, but as complementary resources with distinct phytochemical profiles and different technological, sensory, and functional potential. Their future relevance in nutrition and functional food development will depend on robust human evidence, improved standardisation, species-specific labelling, and careful distinction between traditional dietary use and concentrated nutraceutical formulations.

## Figures and Tables

**Table 1:** Main groups of bioactive constituents identified in *Matricaria chamomilla* L. flowers and their relevance to dietary, nutraceutical, and functional applications [6,32,38,39,49].

Compound Group	Representative Constituents	Properties/Relevance
**Essential oil constituents**	α-Bisabolol, chamazulene, bisabolol oxides A and B	Anti-inflammatory activity; contribution to aroma profile; technological and formulation relevance
**Flavonoids**	Apigenin, luteolin, quercetin	Antioxidant and anti-inflammatory activity; relevance for dietary and nutraceutical applications
**Phenolic acids**	Caffeic acid, chlorogenic acid, ferulic acid	Antioxidant activity and contribution to the overall bioactive potential of chamomile preparations
**Coumarins**	Herniarin, umbelliferone	Phytochemical significance and potential contribution to biological activity
**Polysaccharides and hydrophilic constituents**	Mucilages, hydrophilic fractions	Functional and technological relevance, especially in aqueous preparations and food/supplement matrices

**Table 2:** Comparison of selected phytochemical, sensory, and practical characteristics of German chamomile (*Matricaria chamomilla* L.) and Roman chamomile (*Chamaemelum nobile* (L.) All.) based on literature data.

Feature	German Chamomile	Roman Chamomile	References
**Latin name**	*Matricaria chamomilla* L. (syn. *M. recutita* L.)	*Chamaemelum nobile* (L.) All.	[7,8,50]
**Type of raw material**	Dried flower heads (*Chamomillae flos*)	Dried flower heads (*Chamaemeli romanae flos*)	[7,50]
**Dominant** **compounds**	α-Bisabolol, α-bisabolol oxides, chamazulene/azulene precursors, apigenin and its glycosides	Angelate and tiglate esters, including isobutyl and 2-methylbutyl angelates; lower apigenin content than in German chamomile	[6,7,8,9,50,52]
**Sensory and** **aromatic profile**	Mild, herbal-floral, characteristically “chamomile-like”	More intense and distinctly aromatic; often described as fruity-herbal and slightly bitter	[9,50]
**Most common applications**	Infusions, extracts, phytotherapy, functional foods, cosmetics	Herbal medicine, aromatherapy, cosmetic and aromatic preparations; traditionally associated with digestive comfort	[6,7,8,50]
**Practical** **relevance**	More extensively studied and frequently used in phytotherapy, nutraceuticals, and functional food research	Valued mainly for its ester-rich essential oil profile and aromatic/cosmetic applications	[6,7,8,9]

**Table 3:** Main chamomile-derived forms used as dietary, nutraceutical, and functional ingredients.

Chamomile-Derived Form	Potential Application	Main Advantage	Main Limitation
Dried flower heads [6,9,30]	Herbal teas, infusions, herbal blends	Natural product perception, simple use, traditional consumer acceptance	Limited aroma stability and compositional variability during storage
Aqueous extract [6,30,36,39]	Functional beverages, hydrophilic products	Easy formulation and good compatibility with aqueous systems	Lower extraction efficiency for lipophilic constituents
Ethanolic/hydroalcoholic extract [6,36,39]	Dietary supplements, botanical concentrates, herbal preparations	Higher content of selected bioactive constituents	Formulation and regulatory limitations related to solvent use
Essential oil [6,39]	Flavouring, selected nutraceutical formulations, cosmetics, aromatherapeutic preparations	High aroma intensity and bioactive constituents	Strong sensory impact, dose limitations, and stability concerns
Plant powder/formulation additive [30,36,37]	Supplements, instant mixtures, dry functional formulations	Convenient use, easy dosing, broad formulation potential	Challenges related to stability, dispersibility, and sensory acceptance
Encapsulated extracts or oils [43,44,45,46,47]	Nutraceutical systems, controlled-release formulations, functional matrices	Improved stability, masking of bitterness or excessive aroma	Higher technological complexity and need for formulation optimisation

**Table 4:** Safety considerations for German and Roman chamomile in dietary, nutraceutical, and functional food applications based on clinical reports, herbal safety resources, regulatory documents, and experimental studies [6,9,10,12,13,36,37,38,50,52,107,111,112,113,114,115,116,117,118,119].

Safety Issue	Evidence Base	Level of Concern	Practical Implications
**Asteraceae/Compositae allergy and cross-reactivity**	Clinical reports, case reports, pharmacovigilance, botanical plausibility	Moderate to high in sensitised individuals	Avoid use or use only under medical advice in individuals allergic to ragweed, chrysanthemum, daisies, marigold, or related species
**Severe allergic reactions, including anaphylaxis**	Rare case reports	Low in general population; high in sensitised individuals	Clear labelling and botanical authentication are required to minimise risk
**Interaction with anticoagulant/antiplatelet therapy**	Case reports and pharmacological plausibility	Moderate, especially for concentrated products	Caution with warfarin, aspirin, clopidogrel, perioperative use, and polyherbal supplements
**Sedative/CNS-active agents**	Mainly theoretical, supported by mechanistic/preclinical data	Low to moderate	Caution with benzodiazepines, opioids, alcohol, sedative antidepressants, and other CNS-active agents
**Antidiabetic medications**	Limited human and mechanistic evidence	Low to moderate	Monitor glycaemia when high-dose extracts are used with antidiabetic therapy
**Antihypertensive medications**	Limited human and mechanistic evidence	Low	Present as precautionary issue, especially for concentrated preparations
**Estrogen-like activity/hormone-sensitive conditions**	Limited in vitro and experimental evidence	Low/uncertain	Avoid overstating; consider precaution in hormone-sensitive conditions
**Pregnancy and lactation**	Limited human safety data; regulatory monographs note insufficient evidence	Uncertain; higher for concentrated products	Avoid high-dose extracts, essential oils, and supplements unless medically supervised
**Infants and young children**	Limited formulation- and dose-specific data	Uncertain	Prefer conservative use and avoid concentrated preparations
**Essential oils**	Concentrated exposure; composition-dependent safety	Moderate if ingested or misused	Not equivalent to infusions; require strict dose, purity, and labelling control
**Adulteration/species substitution**	Analytical and botanical quality-control evidence	Moderate	Use authenticated raw material and species-specific labelling
**Contaminants**	Food safety and botanical ingredient risk assessment	Variable, product-dependent	Test for heavy metals, pesticide residues, mycotoxins, microbial contamination, and foreign plant material

## Data Availability

No new data were created or analyzed in this study. Data sharing is not applicable to this article, as this is a review based on previously published literature.

## References

[1] Cory H., Passarelli S., Szeto J., Tamez M., Mattei J. (2018). The Role of Polyphenols in Human Health and Food Systems: A Mini-Review. Front. Nutr..

[2] Fraga C.G., Croft K.D., Kennedy D.O., Tomás-Barberán F.A. (2019). The Effects of Polyphenols and Other Bioactives on Human Health. Food Funct..

[3] Hossain S., Wazed M.A., Asha S., Amin R., Shimul I.M. (2025). Dietary Phytochemicals in Health and Disease: Mechanisms, Clinical Evidence, and Applications—A Comprehensive Review. Food Sci. Nutr..

[4] Zemestani M., Rafraf M., Asghari-Jafarabadi M. (2016). Chamomile Tea Improves Glycemic Indices and Antioxidants Status in Patients with Type 2 Diabetes Mellitus. Nutrition.

[5] Cheimpeloglou K., Adamantidi T., Prokopiou V., Ofrydopoulou A., Christina C., Tsoupras A., Ramawat K.G., Mérillon J.-M. (2026). Natural Bioactives from Chamomile (*Matricaria chamomilla*) with Health-Promoting Properties for Applications in the Pharmaceutical, Cosmetic, and Food Industries. Natural Products.

[6] El Mihyaoui A., Esteves Da Silva J.C.G., Charfi S., Candela Castillo M.E., Lamarti A., Arnao M.B. (2022). Chamomile (*Matricaria chamomilla* L.): A Review of Ethnomedicinal Use, Phytochemistry and Pharmacological Uses. Life.

[7] Singh O., Khanam Z., Misra N., Srivastava M. (2011). Chamomile (*Matricaria chamomilla* L.): An Overview. Pharmacogn. Rev..

[8] Srivastava J.K. (2010). Chamomile: A Herbal Medicine of the Past with a Bright Future. Mol. Med. Rep..

[9] McKay D.L., Blumberg J.B. (2006). A Review of the Bioactivity and Potential Health Benefits of Chamomile Tea (*Matricaria recutita* L.). Phytother. Res..

[10] Mishra S.K., Roy S., Sagar S., Kamdar J.H., Georrge J.J. (2026). Pharmacological and Therapeutic Insights into *Matricaria chamomilla*: A Comprehensive Review. Curr. Pharmacogenomics Pers. Med..

[11] Suryoprabowo S., Rice C., Wang W., Wang Z. (2026). The Advantages of Chamomile (*Matricaria recutita*) Extract for Health: A Review. Explor. Foods Foodomics.

[12] Franke R., Schilcher H. (2005). Chamomile: Industrial Profiles.

[13] Guimarães R., Barros L., Dueñas M., Calhelha R.C., Carvalho A.M., Santos-Buelga C., Queiroz M.J.R.P., Ferreira I.C.F.R. (2013). Nutrients, Phytochemicals and Bioactivity of Wild Roman Chamomile: A Comparison between the Herb and Its Preparations. Food Chem..

[14] Omidbaigi R., Sefidkon F., Kazemi F. (2004). Influence of Drying Methods on the Essential Oil Content and Composition of Roman Chamomile. Flavour Fragr. J..

[15] WHO (1999). Flos Chamomillae. WHO Monographs on Selected Medicinal Plants.

[16] Szymczak K., Krajewska A., Grzyb M., Jodłowska I., Mietlińska K., Bonikowski R. (2026). Upcycling Roman Chamomile Hydrolate and Apple Pomace Agri-Wastes into Sustainable Cosmetic Ingredients. Antioxidants.

[17] Facenda V.L. (2026). Not Your Grandmother’s Chamomile Tea. Tea & Coffee Trade Journal.

[18] Carbone R., Caracciolo F., Di Vita G., D’Amico M., Spina D. (2025). Consumer Trends in the Herbal Tea Market: A Systematic Literature Review. Food Rev. Int..

[19] Bakry A.M., Abbas S., Ali B., Majeed H., Abouelwafa M.Y., Mousa A., Liang L. (2016). Microencapsulation of Oils: A Comprehensive Review of Benefits, Techniques, and Applications. Compr. Rev. Food Sci. Food Saf..

[20] Bouftou A., Aghmih K., Belfadil D., Rezzouq A., Lakhdar F., Lamine M., Gmouh S., Majid S. (2024). Novel Food Preservation Strategy Using Sprayed PVA/Chitosan-Based Coatings Activated by Macroemulsions of Chamomile Essential Oil Adsorbed on Activated Carbon. Int. J. Biol. Macromol..

[21] Tang Y., Zhou Y., Lan X., Huang D., Luo T., Ji J., Mafang Z., Miao X., Wang H., Wang W. (2019). Electrospun Gelatin Nanofibers Encapsulated with Peppermint and Chamomile Essential Oils as Potential Edible Packaging. J. Agric. Food Chem..

[22] Lemberkovics E., Kakasy A., Szöke E., Simái B. (2003). Effect of Extraction Methods on the Composition of Essential Oils. Acta Hortic..

[23] Carocho M., Barreiro M.F., Morales P., Ferreira I.C.F.R. (2014). Adding Molecules to Food, Pros and Cons: A Review on Synthetic and Natural Food Additives. Compr. Rev. Food Sci. Food Saf..

[24] Avallone R., Zanoli P., Puia G., Kleinschnitz M., Schreier P., Baraldi M. (2000). Pharmacological Profile of Apigenin, a Flavonoid Isolated from *Matricaria chamomilla*. Biochem. Pharmacol..

[25] Lopes B.A., Boscardin P.D., Campos P.M. (2023). Development and Characterization of Nanoemulsion Containing Volatile Oil of *Matricaria recutita* L. *Braz*. Arch. Biol. Technol..

[26] Manzanilla-Herrera H.E., Navarro-Moreno L.G., Carvajal-Zarrabal O., Hernández-Sánchez F., Navarro-Mtz A.K., Capataz-Tafur J., Olaide-Olawunmi A., Nolasco-Hipólito C. (2025). Transforming Chamomile (*Matricaria chamomilla*) Infusion into a Fermented Beverage Using Sucrose and Probiotic Lactic Acid Bacteria. Discov. Food.

[27] Caleja C., Barros L., Antonio A.L., Oliveira M.B.P.P., Ferreira I.C.F.R. (2017). A Comparative Study between Natural and Synthetic Antioxidants: Evaluation of Their Performance after Incorporation into Biscuits. Food Chem..

[28] Aamir M., Abid A., Azam I., Ikram A., Saeed F., Afzaal M., Ateeq H., Akram N., Hussain S., Khan M.R. (2024). Characterization of Carbonated Beverage Fortified with Chamomile Herbal Extract. Food Sci. Nutr..

[29] Yangilar F., Yildiz P.O. (2018). Effects of Using Combined Essential Oils on Quality Parameters of Bio-Yogurt. J. Food Process. Preserv..

[30] Kerbab K., Sanah I., Djeghim F., Belattar N., Santoro V., D’Elia M., Rastrelli L. (2025). Nutritional Composition, Physicochemical Properties, Antioxidant Activity, and Sensory Quality of *Matricaria chamomilla*-Enriched Wheat Bread. Foods.

[31] Caleja C., Barros L., Antonio A.L., Ciric A., Barreira J.C.M., Sokovic M., Oliveira M.B.P.P., Santos-Buelga C., Ferreira I.C.F.R. (2015). Development of a Functional Dairy Food: Exploring Bioactive and Preservation Effects of Chamomile (*Matricaria recutita* L.). J. Funct. Foods.

[32] Sotiropoulou N.S., Megremi S.F., Tarantilis P. (2020). Evaluation of Antioxidant Activity, Toxicity, and Phenolic Profile of Aqueous Extracts of Chamomile (*Matricaria chamomilla* L.) and Sage (*Salvia officinalis* L.) Prepared at Different Temperatures. Appl. Sci..

[33] Avula B., Wang Y.-H., Wang M., Avonto C., Zhao J., Smillie T.J., Rua D., Khan I.A. (2014). Quantitative Determination of Phenolic Compounds by UHPLC-UV–MS and Use of Partial Least-Square Discriminant Analysis to Differentiate Chemo-Types of Chamomile/Chrysanthemum Flower Heads. J. Pharm. Biomed. Anal..

[34] Srivastava J. (2009). Extraction, Characterization, Stability and Biological Activity of Flavonoids Isolated from Chamomile Flowers. Mol. Cell. Pharmacol..

[35] Pytlakowska K., Kita A., Janoska P., Połowniak M., Kozik V. (2012). Multi-Element Analysis of Mineral and Trace Elements in Medicinal Herbs and Their Infusions. Food Chem..

[36] Sepp J., Koshovyi O., Jakstas V., Žvikas V., Botsula I., Kireyev I., Tsemenko K., Kukhtenko O., Kogermann K., Heinämäki J. (2024). Phytochemical, Technological, and Pharmacological Study on the Galenic Dry Extracts Prepared from German Chamomile (*Matricaria chamomilla* L.) Flowers. Plants.

[37] Koshovyi O., Sepp J., Jakštas V., Žvikas V., Kireyev I., Karpun Y., Odyntsova V., Heinämäki J., Raal A. (2024). German Chamomile (*Matricaria chamomilla* L.) Flower Extract, Its Amino Acid Preparations and 3D-Printed Dosage Forms: Phytochemical, Pharmacological, Technological, and Molecular Docking Study. Int. J. Mol. Sci..

[38] Catani M.V., Rinaldi F., Tullio V., Gasperi V., Savini I. (2021). Comparative Analysis of Phenolic Composition of Six Commercially Available Chamomile (*Matricaria chamomilla* L.) Extracts: Potential Biological Implications. Int. J. Mol. Sci..

[39] Dai Y.-L., Li Y., Wang Q., Niu F.-J., Li K.-W., Wang Y.-Y., Wang J., Zhou C.-Z., Gao L.-N. (2022). Chamomile: A Review of Its Traditional Uses, Chemical Constituents, Pharmacological Activities and Quality Control Studies. Molecules.

[40] Sharafzadeh S., Alizadeh O. (2011). German and Roman Chamomile. J. Appl. Pharm. Sci..

[41] HMPC (2011). EMA Assessment Report on Chamaemelum nobile (L.) All., Flos.

[42] Polcaro L.M., Cerulli A., Masullo M., Piacente S. (2025). Phytochemical Investigation of *Chamaemelum nobile* L. and Evaluation of Acetylcholinesterase and Tyrosinase Inhibitory Activity. Plants.

[43] Chiș M.S., Păucean A., Man S., Pop A., Mureșan A.E., Fostoc G., Muste S. (2019). A Comprehensive Review Regarding the Botanical Origin, Medicinal Uses and Chemical Composition of Roman and German Chamomile. Hop Med. Plants.

[44] Antonelli A., Fabbri C. (1998). Study on Roman Chamomile (*Chamaemelum nobile* L. All.) Oil. J. Essent. Oil Res..

[45] Colombo F., Restani P., Biella S., Di Lorenzo C. (2020). Botanicals in Functional Foods and Food Supplements: Tradition, Efficacy and Regulatory Aspects. Appl. Sci..

[46] Prakhyath K.M., Sharma P.M., Pragadheesh V.S., Veeragurunathan V., Gopalakrishnan V.A.K., Ghosh A., Yogendra N.D. (2026). Dynamics of Gracilaria Edulis Liquid Extract on Agromorphology, Yield, and Essential Oil Profile of Chamomile (*Matricaria recutita* L.). BMC Plant Biol..

[47] Tai Y., Hou X., Liu C., Sun J., Guo C., Su L., Jiang W., Ling C., Wang C., Wang H. (2020). Phytochemical and Comparative Transcriptome Analyses Reveal Different Regulatory Mechanisms in the Terpenoid Biosynthesis Pathways between *Matricaria recutita* L. and *Chamaemelum nobile* L. BMC Genom..

[48] Batovska D., Panova N., Gerasimova A., Tumbarski Y., Ivanov I., Dincheva I., Yotkovska I., Gentscheva G., Nikolova K. (2025). Chamomile Matters: Species- and Producer-Dependent Variation in Bulgarian *Matricaria recutita* L. and *Chamaemelum nobile* L. Essential Oils and Their Cosmetic Potential. Cosmetics.

[49] Kolodziejczyk-Czepas J., Bijak M., Saluk J., Ponczek M.B., Zbikowska H.M., Nowak P., Tsirigotis-Maniecka M., Pawlaczyk I. (2015). Radical Scavenging and Antioxidant Effects of *Matricaria chamomilla* Polyphenolic–Polysaccharide Conjugates. Int. J. Biol. Macromol..

[50] Formisano C., Delfine S., Oliviero F., Tenore G.C., Rigano D., Senatore F. (2015). Correlation among Environmental Factors, Chemical Composition and Antioxidative Properties of Essential Oil and Extracts of Chamomile (*Matricaria chamomilla* L.) Collected in Molise (South-Central Italy). Ind. Crops Prod..

[51] Ali A., Tabanca N., Raman V., Avanto C., Yang X., Demirci B., Chittiboyina A., Khan I.A. (2023). Chemical Compositions of Essential Oils from German, Roman, and Chinese Chamomile Flowers and Their Biological Activities against Three Economically Important Insect Pests. Rec. Nat. Prod..

[52] Orav A., Raal A., Arak E. (2010). Content and Composition of the Essential Oil of *Chamomilla recutita* (L.) Rauschert from Some European Countries. Nat. Prod. Res..

[53] Popa V.M., Borozan A.B., Dumbrava D.G., Moldovan C., Raba D.N. (2025). Essential Oils and Microbial Load in Yogurt Milk: An Influence Study. Int. Multidiscip. Sci. GeoConf. SGEM.

[54] Sah A., Naseef P.P., Kuruniyan M.S., Jain G.K., Zakir F., Aggarwal G. (2022). A Comprehensive Study of Therapeutic Applications of Chamomile. Pharmaceuticals.

[55] Bekhti N., Fedoul F., Fouzia M., Merazi Y., Piras A., Atma W. (2022). Phenolic Profile and Biological Activities of Extracts of *Matricaria chamomilla* L. from the Western Algeria. Acta Period. Technol..

[56] Molnar M., Mendešević N., Šubarić D., Banjari I., Jokić S. (2017). Comparison of Various Techniques for the Extraction of Umbelliferone and Herniarin in *Matricaria chamomilla* Processing Fractions. Chem. Cent. J..

[57] Petruľová-Poracká V., Repčák M., Vilková M., Imrich J. (2013). Coumarins of *Matricaria chamomilla* L.: Aglycones and Glycosides. Food Chem..

[58] Sándor Z., Mottaghipisheh J., Veres K., Hohmann J., Bencsik T., Horváth A., Kelemen D., Papp R., Barthó L., Csupor D. (2018). Evidence Supports Tradition: The in Vitro Effects of Roman Chamomile on Smooth Muscles. Front. Pharmacol..

[59] Baranauskienė R., Venskutonis P.R., Ragažinskienė O. (2022). Valorisation of Roman Chamomile (*Chamaemelum nobile* L.) Herb by Comprehensive Evaluation of Hydrodistilled Aroma and Residual Non-Volatile Fractions. Food Res. Int..

[60] Filipović V., Marković T., Dimitrijević S., Song A., Prijić Ž., Mikić S., Čutović N., Ugrenović V. (2024). The First Study on Cultivating Roman Chamomile (*Chamaemelum nobile* (L.) All.) for Its Flower and Essential Oil in Southeast Serbia. Horticulturae.

[61] Das S., Horváth B., Šafranko S., Jokić S., Széchenyi A., Kőszegi T. (2019). Antimicrobial Activity of Chamomile Essential Oil: Effect of Different Formulations. Molecules.

[62] Umezu T., Sano T., Hayashi J., Yoshikawa Y., Shibata Y. (2017). Identification of Isobutyl Angelate, Isoamyl Angelate and 2-methylbutyl Isobutyrate as Active Constituents in Roman Chamomile Essential Oil That Promotes Mouse Ambulation. Flavour Fragr. J..

[63] Al-Snafi A.E. (2016). Medical Importance of *Anthemis Nobilis* (*Chamaemelum nobile*)—A Review. Asian J. Pharm. Sci. Technol..

[64] Carnat A., Carnat A.P., Fraisse D., Ricoux L., Lamaison J.L. (2004). The Aromatic and Polyphenolic Composition of Roman Camomile Tea. Fitoterapia.

[65] Tschan G.M., König G.M., Wright A.D., Sticher O. (1996). Chamaemeloside, a New Flavonoid Glycoside from *Chamaemelum nobile*. Phytochemistry.

[66] Ma C., Winsor L., Daneshtalab M. (2007). Quantification of Spiroether Isomers and Herniarin of Different Parts of *Matricaria Matricarioides* and Flowers of *Chamaemelum nobile*. Phytochem. Anal..

[67] HMPC (2015). EMA European Union Herbal Monograph on Matricaria recutita L., Flos.

[68] Cattivelli A., Zannini M., De Angeli M., D’Arca D., Minischetti V., Conte A., Tagliazucchi D. (2024). Bioaccessibility of Flavones, Flavanones, and Flavonols from Vegetable Foods and Beverages. Biology.

[69] Chen P., Chen F., Guo Z., Lei J., Zhou B. (2023). Recent Advancement in Bioeffect, Metabolism, Stability, and Delivery Systems of Apigenin, a Natural Flavonoid Compound: Challenges and Perspectives. Front. Nutr..

[70] Allemailem K.S., Almatroudi A., Alharbi H.O.A., AlSuhaymi N., Alsugoor M.H., Aldakheel F.M., Khan A.A., Rahmani A.H. (2024). Apigenin: A Bioflavonoid with a Promising Role in Disease Prevention and Treatment. Biomedicines.

[71] Sarma N., Upton R., Rose U., Guo D., Marles R., Khan I., Giancaspro G. (2023). Pharmacopeial Standards for the Quality Control of Botanical Dietary Supplements in the United States. J. Diet. Suppl..

[72] Haghi P.B., Mokarram R.R., Khiabani M.S., Hamishekar H., Kafil H.S., Paryad P., Abedi-Firoozjah R., Tavassoli M. (2025). Green Synthesis of Silver Nanoparticles Using Chamomile Extract for Xanthan/Agar and Bacterial Nanocellulose Antimicrobial Nanobiocomposite. J. Food Meas. Charact..

[73] Tripathy S., Srivastav P.P. (2026). Encapsulation of Food Bioactive Compounds Using Electrohydrodynamic Techniques: From Fundamentals to Industrial Applications. Front. Food Sci. Technol..

[74] Mao J.J., Xie S.X., Keefe J.R., Soeller I., Li Q.S., Amsterdam J.D. (2016). Long-Term Chamomile (*Matricaria chamomilla* L.) Treatment for Generalized Anxiety Disorder: A Randomized Clinical Trial. Phytomedicine.

[75] Villa-Rodriguez J.A., Kerimi A., Abranko L., Tumova S., Ford L., Blackburn R.S., Rayner C., Williamson G. (2018). Acute Metabolic Actions of the Major Polyphenols in Chamomile: An in Vitro Mechanistic Study on Their Potential to Attenuate Postprandial Hyperglycaemia. Sci. Rep..

[76] Jabri M.-A., Sakly M., Marzouki L., Sebai H. (2017). Chamomile (*Matricaria Recutita* L.) Decoction Extract Inhibits in Vitro Intestinal Glucose Absorption and Attenuates High Fat Diet-Induced Lipotoxicity and Oxidative Stress. Biomed. Pharmacother..

[77] Salehi B., Venditti A., Sharifi-Rad M., Kręgiel D., Sharifi-Rad J., Durazzo A., Lucarini M., Santini A., Souto E.B., Novellino E. (2019). The Therapeutic Potential of Apigenin. Int. J. Mol. Sci..

[78] Kamatou G.P.P., Viljoen A.M. (2010). A Review of the Application and Pharmacological Properties of *α* -Bisabolol and *α* -Bisabolol-Rich Oils. J. Am. Oil Chem. Soc..

[79] Seyedjavadi S.S., Khani S., Eslamifar A., Ajdary S., Goudarzi M., Halabian R., Akbari R., Zare-Zardini H., Imani Fooladi A.A., Amani J. (2020). The Antifungal Peptide MCh-AMP1 Derived From *Matricaria chamomilla* Inhibits Candida Albicans Growth via Inducing ROS Generation and Altering Fungal Cell Membrane Permeability. Front. Microbiol..

[80] De Oliveira P.R.S., Pretes N.S., Ribeiro A.C., Castro J.C., Garcia F.P., Nakamura C.V., Bona E., Mikcha J.M.G., Junior M.M., De Abreu Filho B.A. (2024). Comparative Assessment of Antibacterial Activity of *Matricaria chamomilla* L. Extract, Nisin and of Its Combination against *Alicyclobacillus* Spp. Food Microbiol..

[81] Ghamchini V.M., Salami M., Mohammadi G.R., Moradi Z., Kavosi A., Movahedi A., Bidkhori M., Aryaeefar M.R. (2019). The Effect of Chamomile Tea on Anxiety and Depression in Cancer Patients Treated with Chemotherapy. J. Young Pharm..

[82] Kato A., Minoshima Y., Yamamoto J., Adachi I., Watson A.A., Nash R.J. (2008). Protective Effects of Dietary Chamomile Tea on Diabetic Complications. J. Agric. Food Chem..

[83] Mehmood M.H., Munir S., Khalid U.A., Asrar M., Gilani A.H. (2015). Antidiarrhoeal, Antisecretory and Antispasmodic Activities of *Matricaria chamomilla* Are Mediated Predominantly through K+-Channels Activation. BMC Complement. Altern. Med..

[84] Forster H., Niklas H., Lutz S. (1980). Antispasmodic Effects of Some Medicinal Plants. Planta Med..

[85] Bezerra S.B., Leal L.K.A.M., Nogueira N.A.P., Campos A.R. (2009). Bisabolol-Induced Gastroprotection Against Acute Gastric Lesions: Role of Prostaglandins, Nitric Oxide, and K^+^_ATP_ Channels. J. Med. Food.

[86] Szelenyi I., Isaac O., Thiemer K. (1979). Pharmakologische Untersuchungen von Kamillen–Inhaltsstoffen—III. Tierexperimentelle Untersuchungen Über Die Ulkusprotektive Wirkung Der Kamille. Planta Med..

[87] Shaaban M., El-Hagrassi A.M., Osman A.F., Soltan M.M. (2022). Bioactive Compounds from *Matricaria chamomilla*: Structure Identification, in Vitro Antiproliferative, Antimigratory, Antiangiogenic, and Antiadenoviral Activities. Z. Für Naturforschung C.

[88] Nikseresht M., Kamali A., Rahimi H., Delaviz H., Toori M., Kashani I., Mahmoudi R. (2017). The Hydroalcoholic Extract of *Matricaria chamomilla* Suppresses Migration and Invasion of Human Breast Cancer MDA-MB-468 and MCF-7 Cell Lines. Pharmacogn. Res..

[89] Chadwick M., Trewin H., Gawthrop F., Wagstaff C. (2013). Sesquiterpenoids Lactones: Benefits to Plants and People. Int. J. Mol. Sci..

[90] Siedle B., García-Piñeres A.J., Murillo R., Schulte-Mönting J., Castro V., Rüngeler P., Klaas C.A., Da Costa F.B., Kisiel W., Merfort I. (2004). Quantitative Structure−Activity Relationship of Sesquiterpene Lactones as Inhibitors of the Transcription Factor NF-κB. J. Med. Chem..

[91] Mathema V.B., Koh Y.-S., Thakuri B.C., Sillanpää M. (2012). Parthenolide, a Sesquiterpene Lactone, Expresses Multiple Anti-Cancer and Anti-Inflammatory Activities. Inflammation.

[92] Aremu O.O., Tata C.M., Sewani-Rusike C.R., Oyedeji A.O., Oyedeji O.O., Nkeh-Chungag B.N. (2019). Phytochemical Composition, and Analgesic and Antiinflammatory Properties of Essential Oil of *Chamaemelum nobile* (Asteraceae L All) in Rodents. Trop. J. Pharm. Res..

[93] Mollova S., Stanev S., Bojilov D., Manolov S., Mazova N., Koleva Y., Stoyanova A. (2024). Chemical Composition and Antioxidant Activity of Roman Chamomile (*Anthemis nobilis* L.) Essential Oil. Nat. Prod. Commun..

[94] Abdoul-Latif M., Ainane A., Oumaskour K., Boujaber N., Mohamed J., Ainane T. (2021). Chemical Composition and Antimicrobial Activity of the Essential Oil of *Chamaemelum nobile* (L.) All. Italo-Lat. Am. Soc. Ethnomed. SILAE.

[95] Aćimović M., Zheljazkov V.D., Šovljanski O.L., Tomić A.M., Cvetković M., Pezo L., Lončar B., Vukić D., Rat M., Vujisić L. (2025). Effect of Distillation Method on the Antimicrobial Potential and Composition of Roman Chamomile (*Chamaemelum nobile* (L.) All.) Essential Oil and Hydrolate. J. Essent. Oil Bear. Plants.

[96] Nazzaro F., Fratianni F., De Martino L., Coppola R., De Feo V. (2013). Effect of Essential Oils on Pathogenic Bacteria. Pharmaceuticals.

[97] Bakkali F., Averbeck S., Averbeck D., Idaomar M. (2008). Biological Effects of Essential Oils—A Review. Food Chem. Toxicol..

[98] Ferreira L.E., Benincasa B.I., Fachin A.L., Contini S.H.T., França S.C., Chagas A.C.S., Beleboni R.O. (2018). Essential Oils of *Citrus aurantifolia*, *Anthemis nobile* and *Lavandula officinalis*: In Vitro Anthelmintic Activities against *Haemonchus contortus*. Parasit. Vectors.

[99] André W.P.P., Ribeiro W.L.C., Oliveira L.M.B.D., Macedo I.T.F., Rondon F.C.M., Bevilaqua C.M.L. (2018). Essential Oils and Their Bioactive Compounds in the Control of Gastrointestinal Nematodes of Small Ruminants. Acta Sci. Vet..

[100] Katiki L.M., Barbieri A.M.E., Araujo R.C., Veríssimo C.J., Louvandini H., Ferreira J.F.S. (2017). Synergistic Interaction of Ten Essential Oils against *Haemonchus contortus* in Vitro. Vet. Parasitol..

[101] Kandelous H.M., Salimi M., Khori V., Rastkari N., Amanzadeh A., Salimi M. (2016). Mitochondrial Apoptosis Induced by *Chamaemelum nobile* Extract in Breast Cancer Cells. Iran. J. Pharm. Res..

[102] Srivastava J.K., Gupta S. (2007). Antiproliferative and Apoptotic Effects of Chamomile Extract in Various Human Cancer Cells. J. Agric. Food Chem..

[103] Zolghadri S., Bahrami A., Hassan Khan M.T., Munoz-Munoz J., Garcia-Molina F., Garcia-Canovas F., Saboury A.A. (2019). A Comprehensive Review on Tyrosinase Inhibitors. J. Enzym. Inhib. Med. Chem..

[104] Murray A., Faraoni M., Castro M., Alza N., Cavallaro V. (2013). Natural AChE Inhibitors from Plants and Their Contribution to Alzheimer’s Disease Therapy. Curr. Neuropharmacol..

[105] Ayaz M., Sadiq A., Junaid M., Ullah F., Subhan F., Ahmed J. (2017). Neuroprotective and Anti-Aging Potentials of Essential Oils from Aromatic and Medicinal Plants. Front. Aging Neurosci..

[106] FDA (2014). Spices and Other Natural Seasonings and Flavorings.

[107] NCCIH (2024). Chamomile: Usefulness and Safety.

[108] Burdock G.A. (2016). Fenaroli’s Handbook of Flavor Ingredients.

[109] Rosol T.J., Cohen S.M., Eisenbrand G., Fukushima S., Gooderham N.J., Guengerich F.P., Hecht S.S., Rietjens I.M.C.M., Davidsen J.M., Harman C.L. (2023). FEMA GRAS Assessment of Natural Flavor Complexes: Lemongrass Oil, Chamomile Oils, Citronella Oil and Related Flavoring Ingredients. Food Chem. Toxicol..

[110] Santini A., Cammarata S.M., Capone G., Ianaro A., Tenore G.C., Pani L., Novellino E. (2018). Nutraceuticals: Opening the Debate for a Regulatory Framework. Br. J. Clin. Pharmacol..

[111] Tang D., Chen K., Huang L., Li J. (2017). Pharmacokinetic Properties and Drug Interactions of Apigenin, a Natural Flavone. Expert Opin. Drug Metab. Toxicol..

[112] Segal R. (2006). Warfarin Interaction with *Matricaria chamomilla*. Can. Med. Assoc. J..

[113] Schwartz J.A., Romeiser J.L., Kimura R., Senzel L., Galanakis D., Halper D., Mena S., Bennett-Guerrero E. (2023). Effect of Chamomile Intake on Blood Coagulation Tests in Healthy Volunteers: A Randomized, Placebo-Controlled, Crossover Trial. Perioper. Med..

[114] Sarecka-Hujar B., Szulc-Musioł B. (2022). Herbal Medicines—Are They Effective and Safe during Pregnancy?. Pharmaceutics.

[115] Ferguson T., Gordon B. (2025). The Efficacy and Safety of Using Chamomile Products During Pregnancy and the Postpartum Period. Cureus.

[116] EFSA (2009). EFSA Scientific Committee Guidance on Safety Assessment of Botanicals and Botanical Preparations Intended for Use as Ingredients in Food Supplements. EFSA J..

[117] Ratajczak M., Kaminska D., Światły-Błaszkiewicz A., Matysiak J. (2020). Quality of Dietary Supplements Containing Plant-Derived Ingredients Reconsidered by Microbiological Approach. Int. J. Environ. Res. Public Health.

[118] Pallarés N., Berrada H., Font G., Ferrer E. (2022). Mycotoxins Occurrence in Medicinal Herbs Dietary Supplements and Exposure Assessment. J. Food Sci. Technol..

[119] Kanabus J., Bryła M., Leśnowolska-Wnuczek K., Waśkiewicz A., Twarużek M. (2025). Mycotoxins Occurrence in Herbs, Spices, Dietary Supplements, and Their Exposure Assessment. Toxins.

[120] Shipkowski K.A., Betz J.M., Birnbaum L.S., Bucher J.R., Coates P.M., Hopp D.C., MacKay D., Oketch-Rabah H., Walker N.J., Welch C. (2018). Naturally Complex: Perspectives and Challenges Associated with Botanical Dietary Supplement Safety Assessment. Food Chem. Toxicol..

[121] Kim N.-C. (2021). Need for Pharmacopeial Quality Standards for Botanical Dietary Supplements and Herbal Medicines. Food Suppl. Biomater. Health.

[122] Heinrich M., Appendino G., Efferth T., Fürst R., Izzo A.A., Kayser O., Pezzuto J.M., Viljoen A. (2020). Best Practice in Research—Overcoming Common Challenges in Phytopharmacological Research. J. Ethnopharmacol..

[123] Gafner S., Blumenthal M., Foster S., Cardellina J.H., Khan I.A., Upton R. (2023). Botanical Ingredient Forensics: Detection of Attempts to Deceive Commonly Used Analytical Methods for Authenticating Herbal Dietary and Food Ingredients and Supplements. J. Nat. Prod..

